# Prediction Model of Distant Metastasis in Oral Cavity Squamous Cell Carcinoma With or Without Regional Lymphatic Metastasis

**DOI:** 10.3389/fonc.2021.713815

**Published:** 2022-01-03

**Authors:** Hsueh-Ju Lu, Yu-Wei Chiu, Wen-San Lan, Chih-Yu Peng, Hsien-Chun Tseng, Chung-Han Hsin, Chun-Yi Chuang, Chun-Chia Chen, Wei-Shiou Huang, Shun-Fa Yang

**Affiliations:** ^1^ Division of Hematology and Oncology, Department of Internal Medicine, Chung Shan Medical University Hospital, Taichung, Taiwan; ^2^ School of Medicine, Chung Shan Medical University, Taichung, Taiwan; ^3^ Department of Dentistry, Chung Shan Medical University Hospital, Taichung, Taiwan; ^4^ School of Dentistry, Chung Shan Medical University, Taichung, Taiwan; ^5^ Institute of Medicine, Chung Shan Medical University, Taichung, Taiwan; ^6^ Department of Radiation Oncology, Chung Shan Medical University Hospital, Taichung, Taiwan; ^7^ Department of Otolaryngology, Chung Shan Medical University Hospital, Taichung, Taiwan; ^8^ Division of Plastic Surgery, Department of Surgery, Chung Shan Medical University Hospital, Taichung, Taiwan; ^9^ Department of Medical Research, Chung Shan Medical University Hospital, Taichung, Taiwan

**Keywords:** oral cavity squamous cell carcinoma, distant metastasis, lymphatic metastasis, prediction model, oral cancer

## Abstract

Patients with oral cavity squamous cell carcinoma (OCSCC) who develop distant metastasis (DM) face poor outcomes, and effective prediction models of DM are rare. A total of 595 patients with OCSCC were retrospectively enrolled in this study. Because pathological N staging significantly influences the development and mechanisms of DM, the patients were divided into nodal-negative (pN−) and -positive (pN+) groups. Clinical outcomes, prognoses, and prediction models were analyzed separately for both groups. Overall, 8.9% (53/595) of these patients developed DM. Among the DM cases, 84.9% (45/53) of them developed DM within the first 3 years. The median overall survival, locoregional recurrence-free survival, time until DM development, and postmetastatic survival were 19.8, 12.7, 14.6, and 4.1 months, respectively. Distinguishing patients who only developed locoregional recurrence from those with DM according to locoregional conditions was difficult. Age, surgical margin, and early locoregional recurrence were predictors of DM that were independent of time until DM in the pN− group; the lymphocyte-to-monocyte ratio, presence of lymphovascular invasion, and early locoregional recurrence in the pN+ group were determined. If one point was scored for each factor, then two scoring systems were used to classify the patients into low- (score = 0), intermittent- (score = 1), or high- (score = 2 or 3) risk for the pN− and pN+ groups. According to this scoring system, the 3-year DM rates for the low, intermittent, and high risk subgroups were 0.0%, 5.9%, and 17.8% for the pN− group and 7.1%, 44.9%, and 82.5% for the pN+ group, respectively. These systems also effectively predicted DM, and the areas under the curve predicted DM occurring within the first 3 years were 0.744 and 0.820 for the pN− and pN+ groups, respectively. In conclusion, effective scoring models were established for predicting DM.

## Introduction

Oral cavity squamous cell carcinoma (OCSCC) is one of the most common types of head and neck squamous cell carcinoma (HNSCC), which is the sixth most common cancer globally and the fourth most common cancer in the Taiwanese male population (1, 2). In addition, 5% to 15% of patients with curative OCSCC develop distant metastasis (DM) during follow-up, for which the prognosis is poor (3–5). The median survival rate is 12.5 mo for those with metastatic HNSCC (6) and 3 mo for those with metastatic OCSCC (4). Thus, metastatic OCSCC research and management is critical.

Unlike locoregional recurrence, for which salvage surgery is a curative option, metastatic OCSCC can generally only be treated with palliative therapies (7–9). Although approved novel agents could prolong survival, most provide no long-term clinical benefit (7–9). The outcomes of DM are also influenced by its clinical presentation; several studies have reported that the number of metastatic lesions significantly influences the survival rates of patients with DM (4, 6). Patients who develop single metastasis or oligometastasis have higher survival rates than do those who develop multiple metastases (4, 6). Unfortunately, most lesions form multiple metastases, with 15% to 30% of cases of DM detected during follow-up for a single metastasis or oligometastasis. Metastatic-direct therapy, such as surgical resection, radiotherapy, and radiofrequency ablation, also influences survival rates, but only cases of single metastasis or oligometastasis are suitable for these aggressive therapy treatments (6). Therefore, the early detection of single and oligometastatic lesions is crucial and influences the outcomes and choice of metastatic-direct therapy.

These single and oligometastatic lesions can be detected through regular screening, but because of the relatively low rate of DM among patients with OCSCC, the cost-effectiveness of regular screening for these patients must be considered. Effective biomarkers that predict DM may provide a suitable method for selecting eligible patients for screening. Several studies have discussed predicting DM (4, 5, 10–14), and pathological neck lymph node involvement was considered to significantly influence the development of DM (15, 16). The primary tumor was long thought to passively permeate the lymphatic system and spread to the regional lymph nodes; the permeated tumor cells would then enter the lymph node vasculature and disseminate to distant organs through the lymphatic system or blood vessel system (17). However, some patients can develop DM without initial pathological neck lymph node involvement (5). The DM model now considers the additional effects of components of primary tumor biology, such as tumor microenvironment, the vascular endothelial growth factor family, and epithelial-mesenchymal transition (17). Primary tumors actively enter the primary tumor lymphatics and primary tumor vasculature simultaneously. The tumor cells then directly metastasize to distant organs through both of these systems. Because the mechanisms of DM can differ between patients with and without neck lymph metastasis, prediction models should be formulated independent of this factor. In addition, though these models should ideally be applied to all patients with OCSCC, only clinicopathological variables are analyzed because these factors can be widely available in clinical settings.

In this study, we enrolled all patients newly diagnosed with OCSCC at Chung Shan Medical University Hospital between 2010 and 2016. Data regarding clinicopathological variables were retrospectively extracted and analyzed. Because pathological N status is highly influential to the DM development (4, 5), the predictors for the patients were analyzed with or without the presence of regional lymph node metastasis. Enrollees were divided into nodal-negative and -positive groups, and the clinical outcomes, prognoses, and the prediction models of these groups were analyzed separately. We hope that these models can be applied in clinical practice, especially in the early detection of DM.

## Methods and Materials

### Study Design, Research Setting, and Patient Selection

This was a single-institute cohort study. Patients newly diagnosed with OCSCC at Chung Shan Medical University Hospital between January 2010 and December 2016 were retrospectively enrolled. The patients were staged according to the American Joint Committee on Cancer staging system (seventh edition) (18) and underwent curative resection at initial diagnosis. At the timing of screening, the patients with locoregional recurrence but who were not newly diagnosed or who experienced secondary primary malignancies were excluded because the factors, such as tumor recurrence and metastatic lesions from secondary primary malignancies, might influence the calculation of time-dependent prognostic and predictive factors. The other exclusion criteria were as follows: patients who did not undergo curative surgery, who received a previous diagnosis and treatment for other HNSCCs, and/or those classified as stage IVC at initial diagnosis. This study was approved by the institutional review board of Chung Shan Medical University Hospital (IRB No. CS2-20050).

### Clinical Characteristics

The clinical data we recorded were the same as those in our previous study and were accessed from the patients’ medical charts (19). We took note of the basic clinicopathological variables included age, sex, primary tumor features (location, staging, and pathological features), and nodal conditions. Adjuvant therapy, included adjuvant chemoradiotherapy, adjuvant radiotherapy, and adjuvant chemotherapy, were also documented. Biochemistry laboratory data documented within 7 d before curative surgery were also compiled. DM-associated information was recorded, such as the numbers and sites of metastatic lesions and their subsequent treatments.

### Classification of Patients With and Without Pathological Neck Lymph Node Involvement

**Figure 1:**
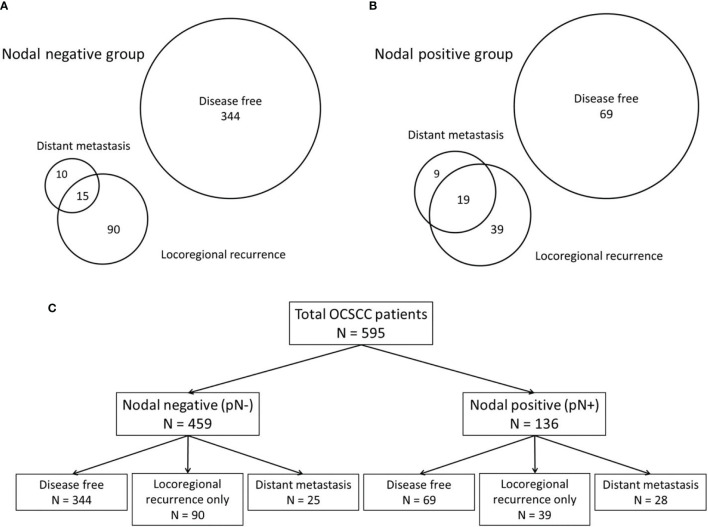
The patients were classified according to their patterns of disease progression. According to the patterns of disease progression, the patients were classified into those with disease-free development (without disease progression), locoregional recurrence only, and distant metastasis with any locoregional status. **(A)** Venn diagram of the nodal-negative group. **(B)** Venn diagram of the nodal-positive group. **(C)** The classifications of both the nodal-negative and -positive groups according to the patterns of disease progression.

Lymphatic and blood vessels are the two primary systems that allow tumor cells to develop into regional metastasis or DM. Because the mechanisms of DM development through the lymphatic or blood vessel systems may differ (17), the phenotypes and predictors are discussed separately herein. Our patients were divided into nodal-negative and -positive groups according to their initial pathological N stage, which was determined in order to identify the influence of regional lymph node metastasis. The patients of both groups were then classified into disease-free status (without disease progression), locoregional recurrence only, and DM with any locoregional status groups to discuss the impact of distant metastasis in clinical outcomes. Prognostic factors and prediction models for DM were separately established for both nodal-negative and -positive groups (**Figure 1**).

### Definition of DM

At the time of writing, monitoring of patients with OCSCC includes imaging studies, such as computed tomography (CT), magnetic resonance imaging (MRI), positron emission tomography-computed tomography, and whole-body bone scanning. Patients should undergo CT and MRI every 3 to 6 mo for the first 2 y and then every 6 to 12 mo in the next 3 to 5 y after curative treatment. PET-CT is conducted when the status of the primary lesion cannot be ascertained *via* CT or MRI. Whole body bone scan (WBBS) should be performed every 6 mo in the first year and then performed again if patients report local bone pain. Here, the imaging studies were evaluated by two independent radiologists; if they could not reach a consensus, then the equivocal patterns were discussed at tumor board conferences.

### Statistical Analysis

Overall survival (OS) was calculated from the date of diagnosis to the date of death or last follow-up. The locoregional recurrence-free survival (LRFS) rate was calculated from the date of disease diagnosis to the date of local or regional recurrence. Time until DM was defined as the date of disease diagnosis until the date of detection of DM. Postmetastatic survival (PMS) was calculated from the date of DM detection to the date of death or the last follow-up.

Correlations between the clinicopathological parameters were analyzed using a χ^2^ or Fisher’s exact test. Cox forward stepwise regression analyses were used to identify the independent factors for PMS and for the time until DM. Only the variables with *P* values of <.05 in univariate analyses were enrolled in the forward multivariate analysis. A two-sided *P* value of <.05 was statistically significant. We utilized the time-dependent receiver operating characteristic (ROC) curve in our prediction models (20). Survival analyses were estimated using the Kaplan-Meier method, and the log-rank test was used to compare the survival curves. SPSS (version 21.0, IBM Corp., Armonk, NY, USA) was used for all statistical analyses.

## Results

### Baseline Characteristics

A total of 595 patients newly diagnosed with OCSCC were retrospectively enrolled in this study. These patients received curative surgical resection. The patients at recurrent stages or with secondary primary malignancies were excluded. In accordance with the initial pathological N staging, the patients were divided into nodal-negative (77.1% [459/595]) and -positive groups (22.9% [136/595]). Mean age of all was 53.9 (28.3-90.3) (nodal-negative group, 53.9 [28.3-90.3]; nodal-positive group, 54.2 [29.2-88.2]) (**Figure S1**). Overall, 8.9% (53/595) of the included patients developed DM during the follow-up period (5.4% [25/459] and 20.6% [28/136] for the nodal-negative and -positive groups, respectively). The basic patient characteristics are presented in **Table 1**.

**Table 1:** Basic characteristics of OCSCC patients with or without distant metastasis.

	pN negative			pN positive		
	Non-distant metastasis N = 434	Distant metastasis N = 25	*P* value	Non-distant metastasis N = 108	Distant metastasis N = 28	*P* value
Age ≥ 65 y	61 (14.1)	8 (32.0)	**0.022**	15 (13.9)	3 (10.7)	0.467
Male	400 (92.2)	22 (88.0)	0.327	95 (88.0)	22 (78.6)	0.164
Personal history						
Smoking	302/431 (70.1)	13/25 (52.0)	**0.050**	72/107 (67.3)	16/28 (57.1)	0.216
Alcohol	252/428 (58.9)	12/25 (48.0)	0.193	74/107 (69.2)	16/28 (57.1)	0.164
Betel nut	294/431 (68.2)	17/25 (68.0)	0.569	74/108 (68.5)	20/28 (71.4)	0.483
Primary tumor site			0.173			0.437
Check mucosa	162 (37.3)	12 (48.0)		41 (38.0)	8 (28.6)	
Oral tongue	136 (31.3)	5 (20.0)		33 (30.6)	8 (28.6)	
Gum	73 (16.8)	4 (16.0)		19 (17.6)	7 (25.0)	
Lip	40 (9.2)	2 (8.0)		8 (7.4)	1 (3.6)	
Mouth floor	13 (3.0)	0 (0.0)		4 (3.8)	1 (3.6)	
Retromolar trigone	5 (1.2)	0 (0.0)		1 (0.9)	1 (3.6)	
Hard palate	2 (0.5)	1 (4.0)		0 (0.0)	1 (3.6)	
Others	3 (0.7)	1 (4.0)		2 (1.9)	1 (3.6)	
Pathologic T staging						
T1-T2	363 (83.6)	16 (64.0)	**0.018**	68 (63.6)	12 (46.2)	0.081
T3-T4	71 (16.4)	9 (36.0)		39 (36.4)	14 (53.8)	
Pathologic N staging			NA			0.203
N0	434 (100.0)	25 (100.0)		0 (0.0)	0 (0.0)	
N1	0 (0.0)	0 (0.0)		47 (43.5)	7 (25.0)	
N2A	0 (0.0)	0 (0.0)		0 (0.0)	0 (0.0)	
N2B	0 (0.0)	0 (0.0)		58 (53.7)	20 (71.4)	
N2C	0 (0.0)	0 (0.0)		3 (2.8)	1 (3.6)	
N3	0 (0.0)	0 (0.0)		0 (0.0)	0 (0.0)	
Pathologic TNM staging			**0.019**			NA
Stage I-II	362 (83.4)	16 (16.6)		0 (0.0)	0 (0.0)	
Stage III-IV	72 (16.6)	9 (36.0)		107 (100)	26 (100)	
Histological grade			0.465			0.550
Well	153 (35.3)	6 (20.0)		9 (8.3)	2 (7.1)	
Moderately	237 (54.6)	18 (72.0)		78 (72.2)	17 (60.7)	
Poorly	32 (7.4)	2 (8.0)		19 (17.6)	8 (28.6)	
Undifferentiated	1 (0.2)	0 (0.0)		0 (0.0)	0 (0.0)	
Unknown	11 (2.5)	0 (0.0)		2 (1.9)	1 (3.6)	
Pathologic feature						
Primary tumor size ≥ 2 cm	198/432 (45.8)	13/25 (52.0)	0.345	78/107 (72.9)	26/28 (92.9)	**0.018**
Extracapsular spread	NA	NA	NA	46/108 (42.6)	11/28 (39.3)	0.463
Depth of invasion ≥ 1 cm	92/394 (23.4)	10/24 (41.7)	**0.042**	43/91 (47.3)	15/21 (71.4)	**0.038**
Lymphovascular invasion	12/405 (3.0)	0/25 (0.0)	0.483	19/103 (18.4)	10/25 (40.0)	**0.024**
Perineural invasion	97/405 (24.0)	7/25 (28.0)	0.400	66/104 (63.5)	19/24 (79.2)	0.108
Surgical margin	264/412 (64.1)	20/25 (80.0)	0.076	74/101 (73.3)	19/26 (73.1)	0.582
Pathologic nodal status						
Lymph node dissection	330/434 (76.0)	18/25 (72.0)	0.400	108/108 (100.0)	28/28 (100.0)	NA
Number of LN dissection ≥15	288/434 (66.4)	17/25 (68.0)	0.528	97/107 (90.4)	23/27 (85.2)	0.301
Positive lymph node ≥ 3	NA	NA	NA	38/107 (35.5)	15/27 (55.6)	**0.047**
Lymph node ratio ≥ 6%	NA	NA	NA	64/107 (59.8)	20/27 (74.1)	0.125
Adjuvant therapy			0.231			0.335
Adjuvant chemoradiotherapy	47 (10.8)	5 (20.0)		50 (46.3)	16 (57.1)	
Adjuvant radiotherapy	51 (11.8)	3 (12.0)		9 (8.3)	0 (0.0)	
Adjuvant chemotherapy	4 (0.9)	1 (4.0)		2 (1.9)	0 (0.0)	
None	332 (76.5)	16 (64.0)		47 (43.5)	12 (42.9)	
Locoregional recurrence < 6 m	17/412 (4.1)	3/24 (12.5)	0.090	11/102 (10.8)	10/26 (38.5)	**0.002**
Preoperative biochemistry data						
White blood count > 10,000/μL	42/400 (10.5)	3/24 (12.5)	0.479	20/102 (19.6)	3/24 (12.5)	0.314
Hemoglobin < 10g/dL	7/400 (1.8)	0/24 (0.0)	0.663	9/102 (8.8)	4/24 (16.7)	0.215
Platelet count > 450,000/μ)	3/400 (0.8)	0/24 (0.0)	0.839	3/102 (2.9)	0/24 (0.0)	0.527
N/L ratio > 2.5	158/370 (42.7)	14/24 (58.3)	0.100	39/98 (39.8)	14/24 (58.3)	0.079
Lymph/Mono < 2.5	25/370 (6.8)	2/24 (8.3)	0.503	9/98 (9.2)	5/24 (20.8)	0.110
Lymph/PLT ratio <0.01	247/369 (66.9)	20/24 (83.3)	0.070	77/98 (78.6)	22/24 (91.7)	0.115

NA, not applicable.

Bold values mean that P value of <.05 was statistically significant.

### Clinical Outcomes

In accordance with disease progression patterns, the patients were classified as disease-free (without disease progression), having locoregional recurrence only, and having DM with any locoregional status. The distributions of the progression patterns were significantly different between the nodal-negative and -positive groups (*P* <.001). The percentage of these three patterns were 72.8% (334/459), 19.6% (90/459), and 5.4% (25/459) in the nodal-negative group, and 50.7% (69/136), 28.7% (39/136), and 20.6% (28/136) in the nodal-positive group (**Figure 1**).

**Figure 2:**
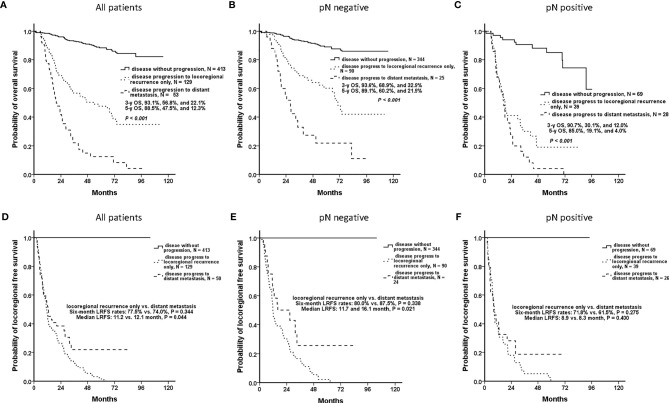
Clinical outcomes of patients who were disease-free, had locoregional recurrence only, and had distant metastasis (DM) with any locoregional status. In accordance with disease progression patterns, the patients were classified as those who were disease-free (without disease progression), who had locoregional recurrence only, and had DM with any locoregional status. **(A)** Overall, the patients who had DM had significantly worsened overall survival (OS) than did the others (*P* <.001). **(B, C)** In both the nodal-negative and -positive groups, the patients who developed DM had significantly poorer OS than those of the patients without DM. **(D)** The patients who developed locoregional recurrence only and DM with any locoregional status had similar 6-mo locoregional recurrence-free survival (LRFS) rates (77.5% and 74.0%, respectively, *P* = .344). It was difficult to differentiate both disease patterns at the early phase. **(E)** For the nodal-negative group, the 6-mo LRFS rates of the patients who developed locoregional recurrence only and who had DM were 80.0% and 87.5%, respectively (*P* = .338), and the median LRFS periods were 11.7 and 16.1 mo, respectively (*P* = .021). **(F)** For the nodal-positive group, the 6-mo LRFS rates were 71.8% and 61.5%, respectively (*P* = .275), and the median LRFS periods were 8.9 and 8.3 mo, respectively (*P* = .400).

For all included OCSCC patients, the OSs of the patients who were disease-free (without disease progression), had locoregional recurrence only, and had DM with any locoregional status were significantly different. 3-y OS of these three patterns were 93.1%, 56.8%, and 22.1%, respectively (*P* <.001), and 5-y OS were 88.5%, 47.5%, and 12.3%, respectively (*P* <.001) (**Figure 2A**). In both the nodal-negative and -positive groups, the patients who developed DM had also significantly poorer OS than those of the patients without DM (**Figures 2B, C**).

However, the patients who developed locoregional recurrence only and DM with any locoregional status had similar 6-mo LRFS rates (77.5% and 74.0%, respectively, *P* = .344) (**Figure 2D**). For the nodal-negative group, the 6-mo LRFS rates of these two progression patterns were 80.0% and 87.5%, respectively (*P* = .338) (**Figure 2E**). And for the nodal-positive group, the 6-mo LRFS rates were 71.8% and 61.5%, respectively (*P* = .275) (**Figure 2F**). Although the patients who developed DM had poor outcomes, it was difficult to distinguish the patients who developed DM or locoregional recurrence only according to the phenotypes of early locoregional recurrence. Effective predictive models are required to ensure that patients at risk of DM undergo regular screening for the early detection of DM.

### Patients Who Developed DM

For the patients who developed DM, the most common metastatic sites were the lung (67.9%), bone (43.4%), and mediastinal lymph node (28.3%). Most metastases (64.2% [34/53]) were multiple metastatic lesions (metastatic lesions ≥ 3). The basic characteristics of the patients who developed DM are listed in **Table 2**.

**Table 2:** The presentations of metastatic lesions.

	Patients with distant metastasis (N = 53)			
		pN negative N = 25	pN positive N = 28	P value
Age ≥ 65 y	11(20.8)	8 (32.0)	3 (10.7)	0.058
Male	44(83.0)	22 (88.0)	22(78.6)	0.295
Personal history				
Smoking	29(54.7)	13(52.0)	16(57.1)	0.460
Alcohol	37(69.8)	12(48.0)	16(57.1)	0.348
Betel nut	28(52.8)	17(68.0)	20(71.4)	0.510
Initial pathologic staging				
pT>2	23/51(43.4)	9/25(36.0)	14/26(53.8)	0.159
pN+	28/53(52.8)	NA	NA	NA
Initial pathologic staging				**<0.001**
Stage I-II	16(31.4)	16(64.0)	0(0.0)	
Stage III-IV	35(68.6)	9(36.0)	26(100)	
Initial histologic type				
Poorly differentiated	10/52 (19.2)	2/25(8.0)	8/27(29.6)	0.050
Initial pathologic feature				
Primary tumor size ≥ 2cm	39/53(73.6)	13/25(52.0)	26/28(92.9)	0.001
Extracapsular spread	11/53(20.8)	0/25(0.0)	11/28(39.3)	<0.001
Depth of invasion >1 cm	25/45(55.6)	10/24(41.7)	15/21(71.4)	0.044
Lymphovascular invasion	10/50(20.0)	0/25(0.0)	10/25(40.0)	<0.001
Perineural invasion	26/49(53.1)	7/25(28.0)	19/24(79.2)	<0.001
Surgical margin	39/51(76.5)	20/25(80.0)	19/26(73.1)	0.401
Adjuvant therapy				**0.017**
Adjuvant chemoradiotherapy	21(39.6)	5(20.0)	16(57.1)	
Adjuvant radiotherapy	3(5.7)	3(12.0)	0(0.0)	
Adjuvant chemotherapy	1(1.9)	1(4.0)	0(0.0)	
None	28(52.8)	16(64.0)	12(42.9)	
Metastatic site				
Lung	36(67.9)	15(60.0)	21(75.0)	0.191
Bone	23(43.4)	14(56.0)	9(32.1)	**0.070**
Mediastinal lymph node	15(28.3)	7(28.0)	8(28.6)	0.603
Pleura	9(17.0)	3(12.0)	6(21.4)	0.295
Liver	6(11.3)	2(8.0)	4(14.3)	0.391
Intra-abdominal organ	3(5.7)	1(4.0)	2(7.1)	0.543
Skin	2(3.8)	2(8.0)	0(0.0)	0.218
Pericardium	1(1.9)	0(0.0)	1(3.6)	0.528
Number of metastatic lesions				0.452
1	13(24.5)	8(32.0)	5(17.9)	
2	6(11.3)	3(12.0)	3(10.7)	
≥3	34(64.2)	14(56.0)	20(71.4)	
Treatment for metastatic lesions				0.285
Palliative chemotherapy	28(52.8)	12(48.0)	16(57.1)	
Local radiotherapy	8(15.1)	6(24.0)	2(7.1)	
Palliative chemotherapy and local radiotherapy	1(1.9)	0(0.0)	1(3.6)	
Best supportive care	15(28.3)	6(24.0)	9(32.1)	
Loss follow up	1(1.9)	1(4.0)	8(15.1)	

Bold values mean that P value of <.05 was statistically significant.

**Figure 3:**
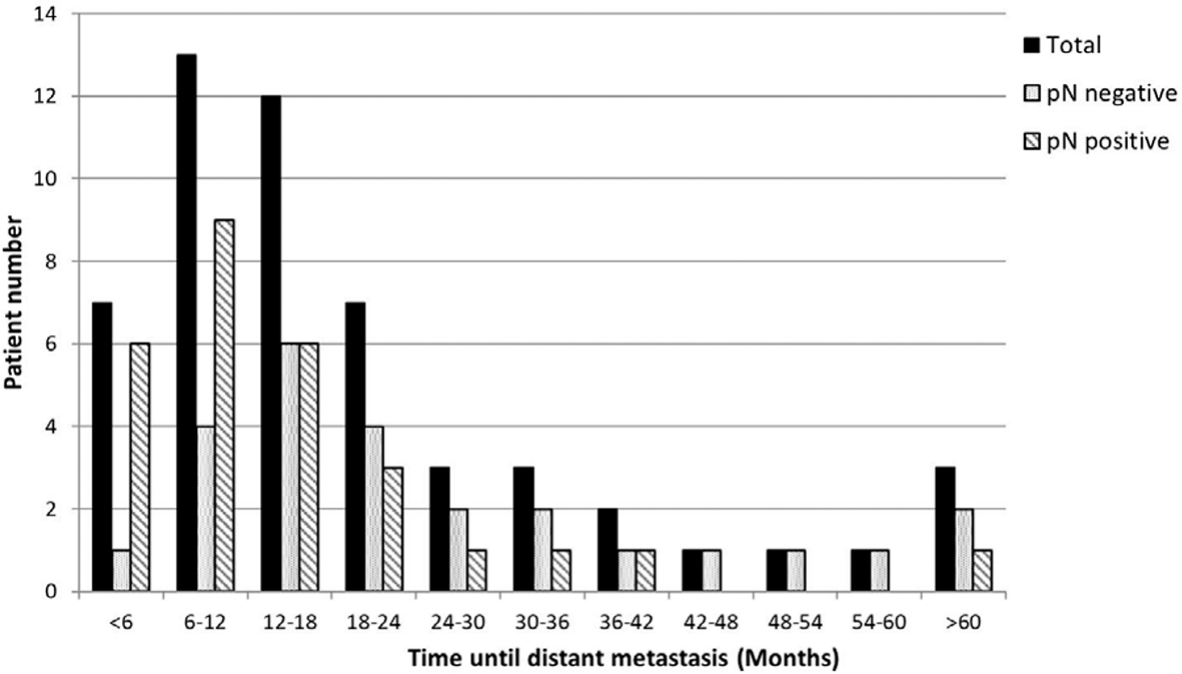
The distribution of time until distant metastasis. Most (84.9%, 45/53) of the DM events occurred within the first 3 y (nodal-negative group, 76% [19/25]; nodal-positive group, 92.9% [26/28]).

The median OS of the patients who developed DM was 19.8 mo (28.0 and 17.3 mo for the nodal-negative and -positive groups, respectively, *P* = .009). The median LRFS period was 12.7 mo (16.1 and 8.3 mo, respectively, *P* = .105). The median time until DM was 14.6 mo (19.2 and 10.8 mo, respectively, *P* = .013). And PMS was only 4.1 mo (5.4 and 3.3 mo, respectively, *P* = .349). Almost 85% (84.9% [45/53]) of the DM events occurred within the first 3 y (nodal-negative group: 76% [19/25]; nodal positive group: 92.9% [26/28]) (**Figure 3**).

Histologically poor differentiation (hazard ratio [HR] 95% confidence interval [CI]: 2.39 [1.13–5.06], *P* = .023) and pleural metastasis (HR [95% CI]: 3.88 [1.67–9.00], *P* = .002) were independent factors for PMS. The number of metastatic lesions (HR [95% CI]: 2.01 [1.04–3.90], *P* = .037) was also a significant factor in the univariate analysis (**Table S1**).

### The Role of Adjuvant Therapy in DM

The distributions of patients receiving adjuvant therapy or not were the same in both the nodal-negative and -positive groups (*P* = .231, and.335, respectively) (**Table 1**). Among the patients with DM, 47.2% (25/53) of them had received adjuvant therapy. The advanced staging (nodal-positive) group had a greater administration of adjuvant therapy than did the lower staging (nodal-negative) group (nodal-positive vs. -negative, 57.1% vs. 36.0%, *P* = .017) (**Table 2**). Although adjuvant therapy did not impact time until DM in statistics, it seemed to be a trend that adjuvant therapy decreased DM occurrence in the nodal-positive group (HR: 0.869, *P* = .694) (**Table S2**).

### DM Prediction Model for Patients With OCSCC

Prediction models for DM were established separately for the nodal-negative and -positive groups because their DM development processes may have differed. In the nodal-negative group, ages greater than 65 y (HR [95% CI]: 3.78 [1.51–9.44], *P* = .004), surgical margin of less than 5 mm (HR [95% CI]: 3.15 [1.06–9.35], *P* = .038), and a locoregional recurrence of less than 6 mo (HR [95% CI]: 7.03 [2.02–24.50], *P* = 0.002) were independent factors for the time until DM. Lymphovascular invasion (HR [95% CI]: 2.81 [1.01–7.86], *P* = .048), a locoregional recurrence of less than 6 mo (HR [95% CI]: 24.35 [8.00–74.11], *P* <.001), and a lymphocyte-to-monocyte ratio of less than 2.5 (HR [95% CI]: 5.38 [1.33–21.72], *P* = .018) were independent factors for the nodal-positive group (**Table S2**). The calculation for the Akaike information criterion of the independent factors is in **Table S3**.

**Figure 4:**
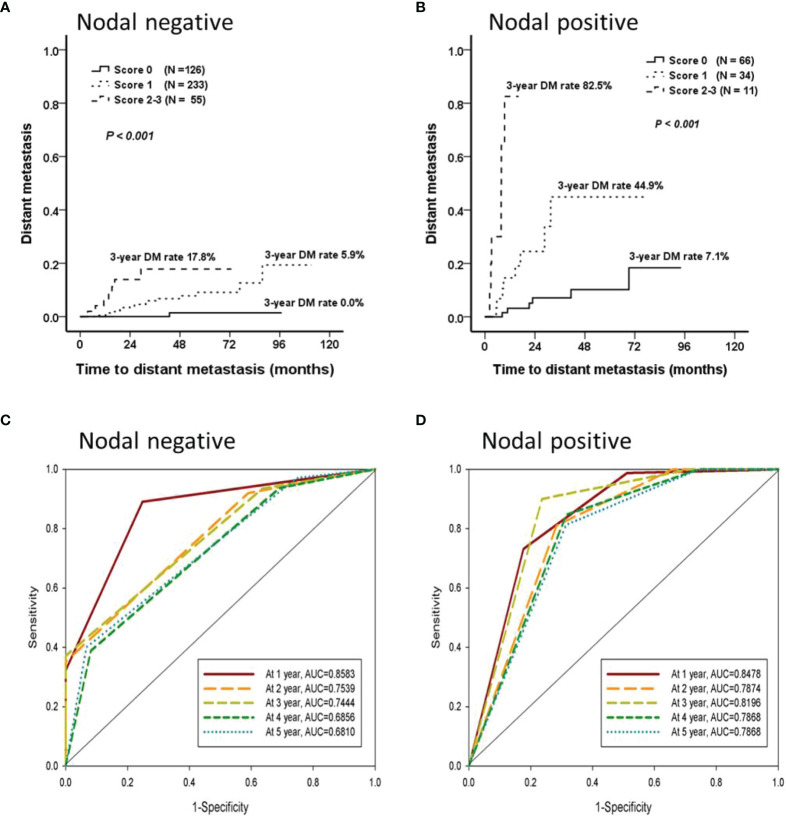
The prediction model for distant metastasis. Cox forward stepwise regression analyzes were used to identify the independent factors for the time until DM. Age, surgical margin, and early locoregional recurrence were predictors of DM that were independent of time until DM in the nodal-negative group; the lymphocyte-to-monocyte ratio, presence of lymphovascular invasion, and early locoregional recurrence in the nodal-positive group were determined. Each independent factor was scored 1 point, and two predictive models could classify patients from both the nodal-negative and -positive groups into low- (score 0), intermittent- (score 1), or high- (score 2 or 3) risk groups separately. **(A, B)** The nodal-negative and -positive 3-y DM rates were 0.0%, 5.9%, and 17.8% and 7.1%, 44.9%, and 82.5%, respectively. **(C, D)** These models were effective predictors of DM events occurring within the first 3 y. The areas under the curve (AUCs) of the nodal-negative and -positive models were 0.744 and 0.820, respectively.

Each independent factor was scored 1 point, and two predictive models were established separately for the nodal-negative and -positive groups. Both models could classify patients into low- (score 0), intermittent- (score 1), and high- (score 2 or 3) risk groups. And 3-y DM rate of these three risk groups were 0.0%, 5.9%, and 17.8% in the nodal-negative group, and 7.1%, 44.9%, and 82.5% in the nodal-positive group, respectively (**Figures 4A, B**). In addition, these models were found to be effective predictors of DM events. The areas under the curve (AUCs) that predicted DM occurring within the first 1 y were 0.858 and 0.848 for the nodal-negative and -positive models, respectively. In addition, up to 85% of the DM events occurred within the first 3 y, and the AUCs predicted this event were 0.744 and 0.820 for both two groups, respectively (**Figures 4C, D**). The AUC, sensitivity, and specificity of each score are presented in **Table 3**.

**Table 3:** Areas under the curve (AUCs), sensitivity, and specificity of the score models to predict distant metastasis.

	pN negative group			pN positive group		
	AUC	Sensitivity	Specificity	AUC	Sensitivity	Specificity
DM within the first 1 year	0.858			0.848		
Score ≥1		100.0%	32.3%		82.3%	73.1%
Score ≥2		75.1%	89.0%		48.8%	98.7%
Score ≥3		0.0%	100.0%		0.0%	100.0%
DM within the first 2 years	0.754			0.787		
Score ≥1		100.0%	35.1%		71.6%	81.0%
Score ≥2		41.0%	92.0%		34.1%	100.0%
Score ≥3		0.0%	100.0%		0.0%	100.0%
DM within the first 3 years	0.744			0.820		
Score ≥1		100.0%	37.1%		76.3%	90.0%
Score ≥2		36.4%	93.5%		28.5%	100.0%
Score ≥3		0.0%	100.0%		0.0%	100.0%
DM within the first 4 years	0.686			0.787		
Score ≥1		91.8%	38.9%		68.5%	84.6%
Score ≥2		30.9%	93.6%		25.6%	100.0%
Score ≥3		0.0%	100.0%		0.0%	100.0%
DM within the first 5 years	0.681			0.787		
Score ≥1		93.3%	40.0%		68.5%	81.3%
Score ≥2		25.4%	97.0%		25.6%	100.0%
Score ≥3		0.0%	100.0%		0.0%	100.0%

## Discussion

A total of 595 patients with OCSCC were retrospectively enrolled in this study. Overall, 8.9% of the included patients developed DM during the follow-up period, with 85% of DM events occurring within the first 3 y following the date of initial diagnosis. Among the patients who developed DM, the median OS, LRFS, time until DM, and PMS were 19.8, 12.7, 14.6, and 4.1 mo, respectively. The lung, bone, and mediastinal lymph nodes were the most common metastatic sites. Histologically poor differentiation and pleural metastasis were independent factors of PMS. Because the phenotypes of locoregional recurrence between the patients who developed DM or locoregional recurrence only were similar, DM was difficult to detect early. Two scoring models predicting DM development were established to distinguish the influence of regional lymph node metastasis. These models could predict DM events occurring within the first 3 y after diagnosis for each patient in the nodal-negative and positive groups. The AUCs of these two models were 0.744 and 0.820, respectively.

In addition, although the mechanism between aging and cancer metastasis in OCSCC was unknown, the results showed that aging influenced the occurrence of DM for both the nodal-negative and -positive groups. In our study, age ≥ 80 y was an independent factor for time until DM in univariant Cox regression for all patients (HR [95% CI]: 8.880 [2.579–30.581], *P* = .001). For the nodal-negative group, ages between 70–80 and ≥ 80 y were significant (ages between 70–80 y, HR [95% CI]: 5.0668 [1.811–14.182], *P* = .002; ages ≥ 80 y, HR [95% CI]: 8.890 [1.056–74.856], *P* = .044). An age ≥ 80 y was also independent (HR [95% CI]: 8.656 [1.731–43.292], *P* = .009) for the nodal-positive group (**Table S3**). Several studies have reported that extracellular matrix degradation may be the reason why cancer metastasis occurred more in elderly patients than in younger patients (21, 22). Future studies are warranted for OCSCC in this issue.

In Taiwan, up to 70% of HNSCC cases are OCSCC (2), and the PMS of patients with OCSCC was significantly poorer than that of non-OCSCC patients. One study has reported that the proportions of patients with cancer of the oral cavity, oropharynx, hypopharynx, and larynx who achieve 2-y PMS are 8.9%, 33.3%, 12.1%, and 21.1%, respectively (*P* <.001) (23). Thus, although the DM rate is lower among patients with OCSCC than among those without OCSCC (14, 24), predicting and managing DM in patients with OCSCC is a major challenge in clinical practice.

Although the shortened period of locoregional control (locoregional recurrence < 6 mo) was found to be independent of time until DM, it was initially difficult to identify the patients who would develop DM; the phenotypes and 6-mo LRFS rate were not significantly different between the patients who developed DM and those with locoregional recurrence only. According to Allen et al. (17), primary tumors actively enter both primary tumor lymphatics and primary tumor vasculature simultaneously and disseminate to distant organs through the lymphatic and blood vessel systems. For locoregional conditions, the mechanisms of tumor-induced lymphangiogenesis and lymph node metastasis have been widely discussed. These mechanisms include the expression of VEGFs, angiopoietins, insulin-like growth factors, and fibroblast growth factors (25), some of which have salient roles in the development of DM (26, 27). Detailed molecular analyses on this topic are warranted in the future.

In addition to locoregional recurrence, DM may develop directly from the primary tumor. In our study, almost 85% of DM events occurred within the first 3 y for the nodal-negative and -positive groups, as reported in other studies (4, 5). The time until DM development was unrelated to nodal status, and the DM process did not depend solely on lymphatic drainage. Metastatic tumor cells enter the primary tumor vasculature directly from the primary tumor lesions (17). These metastatic tumor cells are aggressive and invasive; compared with the genetic profiles of the primary tumor lesions, those of the matched lesions indicated enrichment in hypoxia, angiogenesis, EMT, and glycolysis (12). Other metastatic-related functions, such as cell differentiation, extracellular matrix organization, tissue development, adhesion, immune response, and cancer metabolism, have also been reported (28, 29). The molecular signals of DM differ from those of locoregional recurrence in that the process of DM development does not depend on lymph node metastasis.

Several studies investigated DM predictors on the basis of clinicopathological parameters (10, 11). Hosni et al. found that 14.1% (63/447) of patients with OCSCC in their study were diagnosed with DM during follow-up. Pathological N2 or N3 (pN2 or pN3) and histological grade 2 or 3 (G2–3) were independent factors for DM. However, only patients who received curative surgery followed by adjuvant chemotherapy or chemoradiotherapy were enrolled in this study (10). In addition, Huang et al. classified 312 patients with OCSCC into high-, intermittent-, and low-risk groups according to their human papillomavirus (HPV) viral loads, pN2 status, and tumor depth. The 5-y DM rates for these three groups were 74%, 17%, and 1% (*P* <.001), respectively, and the concordance index was 0.78 (11). Although the model effectively predicted DM in patients with OCSCC, only 5.4% (17/312) of these patients were classified as high-risk with subsequent intensive treatments and follow-up strategies. In our study, patients were divided into nodal-negative and -positive groups, with predictive models established separately for each group. In addition to the shortened interval of locoregional control, other independent factors were related to cancer metastasis, such as age, surgical margin, and lymphovascular invasion status (30–32). In the nodal-negative group, although only 5.4% (25/459) of the patients developed DM during the follow-up period, most (77.1% [459/595]) of the patients with OCSCC were in this group, and this first predictive model was established for them. By contrast, for the nodal-positive group, almost 40.5% (45/111) of the patients were classified as intermittent- to high-risk, and the 3-y DM rates were 44.9% to 82.5%. Both models effectively predicted the development of DM (AUCs predicted DM occurring within the first 3 y, with 0.744 and 0.820 for the nodal-negative and -positive groups, respectively).

In this study, we hoped to provide flexible models to predict DM in clinical practice. Only the parameters which were available in the clinical setting were enrolled for analysis. The patients who missed the score factors would be eliminated in prediction models. The weight of each score factor was different, which might affect the power of the prediction models. In the future, we hoped that artificial intelligence, such as machine learning and deep learning, could be involved in the development of these prediction models.

This study had some limitations. First, although we established a predictive model for DM development, only a single-institute analysis was undertaken; the validation of our results with a large population is required. Second, DM was detected on the basis of image diagnosis and not from autopsy results. Micrometastatic lesions may therefore not have been detected and could constitute missed diagnoses. Third, although molecular information, such as HPV infection status, is vital for predicting DM development in patients with OCSCC (11), only the information available in the patients’ medical charts were analyzed.

## Conclusion

DM development may occur directly through both the lymphatic and blood vessel systems, though the DM development mechanisms of these two systems may differ. In this study, we established two scoring models that could effectively predict DM events within the first 3 y following diagnosis of each patient in both nodal-negative and -positive groups. A validation study is required to verify our findings.

## Data Availability Statement

The raw data supporting the conclusions of this article will be made available by the authors, without undue reservation.

## Ethics Statement

The studies involving human participants were reviewed and approved by Institutional Review Board of Chung Shan Medical University Hospital. The patients/participants provided their written informed consent to participate in this study.

## Author Contributions

H-JL and S-FY proposed the study concept and study design. H-JL edited the manuscript, and S-FY approved the final version to be published. H-JL and W-SL participated in the data collection, statistical analysis, and manuscript preparation. H-JL, Y-WC, C-YP, H-CT, C-HH, C-YC, and W-SH contributed to the collection of clinical information and data processing. All of the authors agree to be accountable for all aspects of the work.

## Funding

This study was supported by research grants to H-JL from the Chung Shan Medical University Hospital, Taiwan (CSH-2020-A-016 and CSH-2020-D-009).

## Conflict of Interest

The authors declare that the research was conducted in the absence of any commercial or financial relationships that could be construed as a potential conflict of interest.

## Publisher’s Note

All claims expressed in this article are solely those of the authors and do not necessarily represent those of their affiliated organizations, or those of the publisher, the editors and the reviewers. Any product that may be evaluated in this article, or claim that may be made by its manufacturer, is not guaranteed or endorsed by the publisher.
