# Supplementary material for: Prediction Model of Distant Metastasis in Oral Cavity Squamous Cell Carcinoma With or Without Regional Lymphatic Metastasis
Source: Front Oncol. 2022 Jan 3;11:713815. doi: 10.3389/fonc.2021.713815 (PMC8761851; doi:10.3389/fonc.2021.713815)
Supplement: Supplementary file 1 [file Table_1.pdf]

Table S1. Univariate and multivariate Cox regression analysis for post-metastatic survival

	Univariate	Multivariate
Age ≥ 65	0.814, 0.918(0.450-1.872)	
Male	0.233, 1.614(0.735-3.545)	
Personal history		
Smoking	0.522, 1.215(0.669-2.204)	
Alcohol	0.749, 0.908(0.505-1.634)	
Betel nut	0.487, 1.255(0.661-2.382)	
Initial pathologic staging		
pT>2	0.419, 1.283(0.701-2.350)	
pN+	0.352, 1.327(0.731-2.406)	
Initial pathologic staging		
Stage III-IV	0.219, 1.522(0.778-2.977)	
Initial histologic type		
Poorly differentiated	**0.044, 2.130(1.020-4.447)**	**0.023, 2.392(1.130-5.062)**
Initial pathologic feature		
Primary tumor size ≥ 2cm	0.791, 0.913(0.467-1.786)	
Extracapsular spread	0.358, 1.415(0.676-2.962)	
Depth of invasion >1 cm	0.933, 0.972(0.502-1.882)	
Lymphovascular invasion	0.955, 1.023(0.471-2.233)	
Perineural invasion	0.610, 1.176(0.631-2.191)	
Surgical margin	0.955, 1.021(0.493-2.115)	
Adjuvant therapy	0.619, 0.861(0.478-1.551)	
Metastatic site		
Lung	0.939, 0.975(0.506-1.878)	
Bone	0.845, 0.943(0.521-1.704)	
Mediastinal lymph node	0.237, 1.523(0.758-3.057)	
Pleura	**0.003, 3.467(1.532-7.848)**	**0.002, 3.881(1.672-9.008)**
Liver	0.668, 1.228(0.481-3.133)	
Intra-abdominal organ	0.302, 1.881(0.561-6.241)	
Skin	0.844, 0.866(0.207-3.624)	
Pericardium	0.180, 4.063(0.524-31.486)	
Number of metastatic lesions ≥ 3	**0.037, 2.018(1.042-3.906)**	

Table S2. Univariate and multivariate Cox regression analysis for time until distant metastasis

	pN negative		pN positive	
	Univariate	Multivariate	Univariate	Multivariate
Age ≥ 65	**0.002, 3.855(1.644-9.041)**	**0.004, 3.782(1.515-9.442)**	0.921, 0.941(0.284-3.124)	
Male	0.284, 0.516(0.154-1.732)		0.209, 0.560(0.227-1.383)	
Personal history				
Smoking	0.195, 0.590(0.265-1.312)		0.212, 0.621(0.293-1.313)	
Alcohol	0.477, 0.749(0.337-1.662)		0.197, 0.610(0.289-1.291)	
Betel nut	0.893, 1.061(0.449-2.508)		0.908, 1.050(0.461-2.389)	
Primary tumor site				
Check mucosa	0.306, 1.507(0.688-3.303)		0.164, 0.558(0.245-1.268)	
Oral tongue	0.232, 0.550(0.206-1.465)		0.831, 1.094(0.481-2.487)	
Pathologic staging				
pT>2	**0.012, 2.841(1.253-6.442)**		**0.002, 3.467(1.561-7.702)**	
pN2/3	NA		0.048, 2.375(1.007-5.604)	
staging III/IV	**0.012, 2.841(1.253-6.442)**		NA	
Poorly differentiated	0.873, 1.125(0.264-4.791)		0.151, 1.837(0.802-4.207)	
Pathologic feature				
Primary tumor size ≥ 2cm	0.495, 1.314(0.599-2.880)		**0.017, 5.752(1.361-24.309)**	
Extracapsular spread	NA		0.677, 1.176(0.548-2.524)	
Depth of invasion >1 cm	**0.031, 2.451(1.086-5.532)**		**0.022, 3.032(1.172-7.841)**	
Lymphovascular invasion	0.603, 0.048(0.000-4600.369)		**0.006, 3.104(1.377-6.997)**	**0.048, 2.818(1.010-7.867)**
Perineural invasion	0.574, 1.284(0.536-3.075)		0.093, 2.330(0.868-6.251)	
Surgical margin	**0.048, 2.693(1.009-7.191)**	**0.038, 3.154(1.063-9.357)**	0.624, 1.242(0.522-2.956)	
Pathologic nodal status				
Lymph node dissection	0.406, 0.691(0.288-1.655)		NA	
Number of LN dissection≥15	0.907, 0.951(0.410-2.205)		0.416, 0.643(0.222-1.862)	
Positive lymph node ≥ 3	NA		**0.006, 2.910(1.349-6.277)**	
Lymph node ratio ≥ 6%	NA		0.129, 1.950(0.824-4.615)	
Adjuvant therapy	0.223, 1.662(0.734-3.762)		0.694, 0.860(0.405-1.826)	
Locoregional recurrence < 6m	**0.005, 5.623(1.664-18.997)**	**0.002, 7.037(2.021-24.500)**	**<0.001, 17.377(7.229-41.772)**	**<0.001, 24.353(8.001-74.119)**
Preoperative biochemistry data				
White blood count > 10,000/μl	0.658, 1.314(0.392-4.407)		0.875, 0.907(0.269-3.054)	
Hemoglobin < 10g/dl	0.684, 0.049(0.00-(1.0E+05))		0.140, 2.247(0.767-6.584)	
Platelet count > 450,000/ μl	0.813, 0.049(0.00-(3.5E+09))		0.636, 0.048(0.000-13744.74)	
N/L ratio > 2.5	0.137, 1.851(0.821-4.172)		**0.016, 2.744(1.211-6.218)**	
Lymph/Mono < 2.5	0.479, 1.693(0.394-7.272)		**0.010, 3.738(1.366-10.232)**	**0.018, 5.386(1.335-21.726)**
Lymph/PLT ratio <0.01	0.137, 2.258(0.771-6.615)		0.167, 2.776(0.652-11.812)	

Table S3. Akaike information criterion (AIC) of the independent factors for time until distant metastasis

	Independent factors	AIC
pN negative group	Age ≥ 65	241.62
	Surgical margin	238.30
	Locoregional recurrence < 6m	246.61
pN positive group	Lymphovascular invasion	114.16
	Locoregional recurrence < 6m	118.25
	Lymph/Mono < 2.5	115.82

Table S4. Univariate Cox regression analysis of specific age group for time until distant metastasis

	Total	pN negative	pN positive
Age			
< 50	1 (reference)	1 (reference)	1 (reference)
< 60 and ≥ 50	0.784, 1.093(0.578-2.068)	0.860, 1.096(0.397-3.025)	0.964, 1.019(0.449-2.312)
< 70 and ≥ 60	0.219, 0.540(0.203-1.441)	0.569, 0.637(0.135-3.011)	0.086, 0.329(0.092-1.170)
< 80 and ≥ 70	0.146, 1.899(0.801-4.504)	**0.002, 5.068(1.811-14.182)**	0.359, 0.039(0.000-40.091)
≥ 80	**0.001, 8.880(2.579-30.581)**	**0.044, 8.890(1.056-74.856)**	**0.009, 8.656(1.731-43.292)**

Figure S1

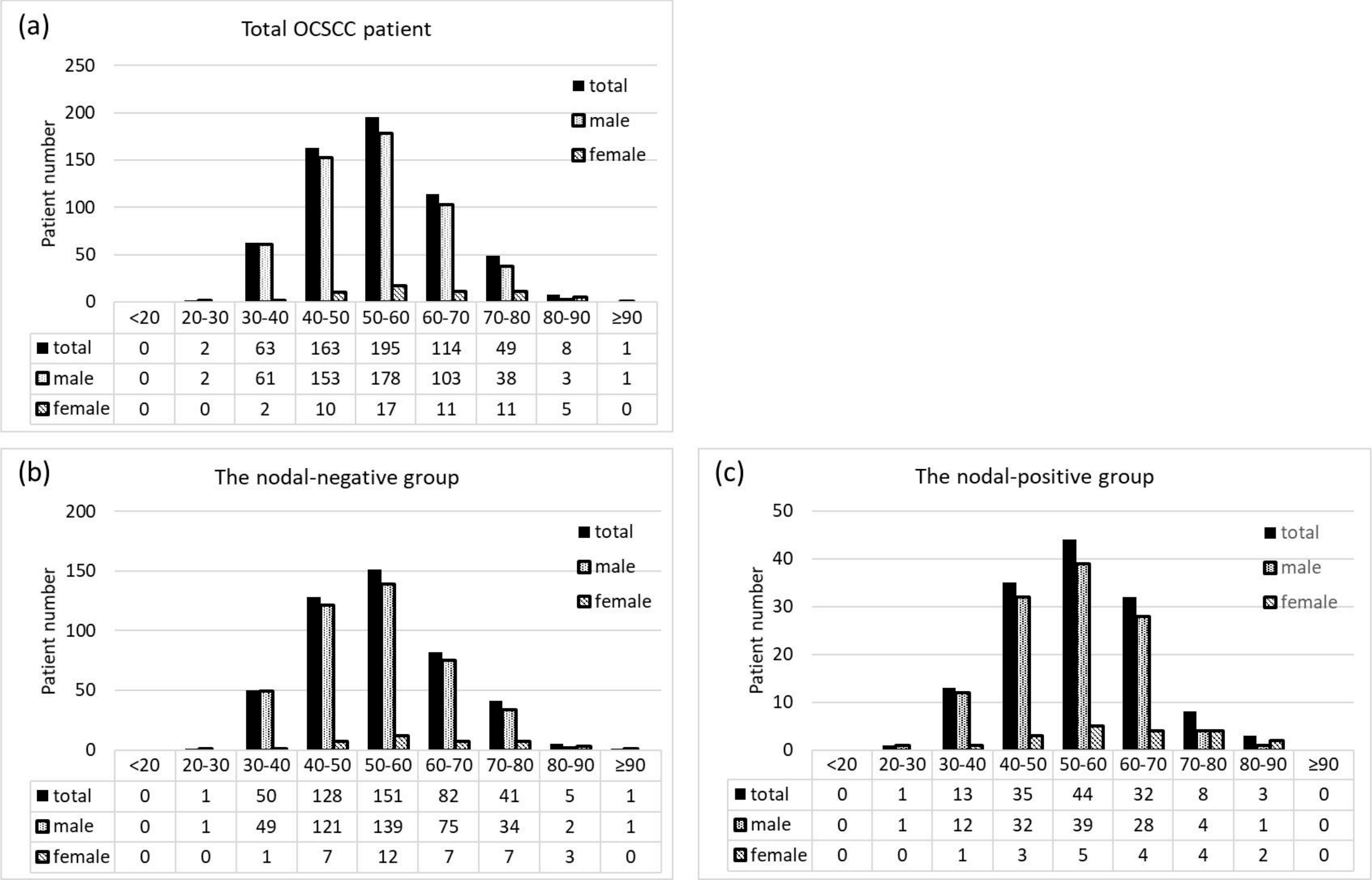

(a) Total OCSCC patient

	<20	20-30	30-40	40-50	50-60	60-70	70-80	80-90	≥90
total	0	2	63	163	195	114	49	8	1
male	0	2	61	153	178	103	38	3	1
female	0	0	2	10	17	11	11	5	0

(b) The nodal-negative group

	<20	20-30	30-40	40-50	50-60	60-70	70-80	80-90	≥90
total	0	1	50	128	151	82	41	5	1
male	0	1	49	121	139	75	34	2	1
female	0	0	1	7	12	7	7	3	0

(c) The nodal-positive group

	<20	20-30	30-40	40-50	50-60	60-70	70-80	80-90	≥90
total	0	1	13	35	44	32	8	3	0
male	0	1	12	32	39	28	4	1	0
female	0	0	1	3	5	4	4	2	0

Figure S1. The distribution of age (a) total OCSCC patient, (b) the nodal-negative group, (c) the nodal-positive group.
